# Face beauty or soul beauty? The influence of facial attractiveness and moral judgment on pain empathy

**DOI:** 10.3389/fpsyg.2022.990637

**Published:** 2022-10-06

**Authors:** Jiawen Zhu, Zhou Yang, Ruining Ma, Lixing Yin, Xucong Hu

**Affiliations:** Key Laboratory of Cognition and Personality, Faculty of Psychology, Southwest University, Chongqing, China

**Keywords:** facial attractiveness, moral judgment, pain empathy, pain intensity, self-uncomfortableness

## Abstract

Previous studies indicated that both facial attractiveness (face beauty) and moral judgment (soul beauty) would impact the responses to others’ pain, however, the effects from facial attractiveness were in controversial. Furthermore, whether facial attractiveness would increase or decrease the effects of moral judgment on pain empathy were still unknown. Based on the videos in which actors with high versus low facial attractiveness under pain or non-pain conditions, study 1 recruited 26 undergraduates to assess the effects of facial attractiveness on participants’ pain intensity rating scores. Then study 2 recruited 85 undergraduates to examine the effects of facial attractiveness and moral judgment on pain empathy by assessing pain intensity and self-uncomfortableness rating scores. Study 1 found that participants rated higher pain intensity scores to actors with high facial attractiveness compared to low facial attractive actors under pain condition. Study 2 found that participants showed higher pain empathic responses for actors with high moral judgment, no matter their facial attractiveness were high or low. For actors with low moral judgment under pain condition, participants showed higher pain empathy to those with high facial attractiveness compared to those with low facial attractiveness. In conclusion, facial attractiveness could facilitate the empathy responses for other’s pain. High facial attractiveness would increase the pain empathic responses to individuals with low moral judgment, however, low facial attractiveness would not decrease the pain empathic responses to individuals with high moral judgment.

## Introduction

Pain empathy triggers pro-social behaviors that allow individuals to receive appropriate care. Pain empathy can increase interpersonal connection, which is of great significance to human survival and development, and can also promote social stability and harmony (Jackson et al., 2006; Chen et al., 2015). Pain empathy is defined as shared perceptions, judgments and emotional representations of others’ pain (Danziger et al., 2006), which includes cognitive component (i.e., perception of other’s pain measured by rating pain intensity scores about other’s pain) and affective component (i.e., self-uncomfortableness of other’s pain assessed by rating self-uncomfortableness scores of other’s pain) (Gladstein, 1983; Shamay-Tsoory, 2011; Giummarra et al., 2015; Kogler et al., 2020). People’s empathic responses to pain are influenced by how they perceive and value others. The external aspects, such as facial attractiveness (Kopiś et al., 2020; Meng et al., 2020), and internal personality traits, such as morality (Fang et al., 2016; Li et al., 2018), are important factors that affect empathic responses to pain.

Facial attractiveness played a critical role in pain empathic responses (Kopiś et al., 2020). Decades of research have proved the effect of “beauty is good,” (Dion et al., 1972) which means that people will attribute more positive traits (Shtudiner, 2020; Shtudiner and Klein, 2020; Ong, 2022) and generate more pro-social behaviors such as trust, cooperation and pain empathy to physically attractive people (Chen et al., 2012; Balconi et al., 2020; Ch’ng, 2021). Some studies indicated that high facial attractiveness would elicit more empathetic responses than low facial attractiveness by using ERP or fMRI methods (Jankowiak-Siuda et al., 2015; Balconi et al., 2020; Meng et al., 2020). For example, in Meng et al. (2020) study, a needle or a cotton swab was placed on the left cheek of a face with neutral expression and participants were asked to rate the pain intensity scores. The results found that those more attractive faces elicited stronger pain empathic responses in N170 and P2 components. However, other studies showed the inconsistent findings. For example, Jankowiak-Siuda et al. (2019) found that participants rated lower pain intensity to high facial attractive actors and rated higher pain intensity to low facial attractive actors. Thus, the effects of facial attractive on pain empathy are still in controversial.

As an important internal personality trait, moral judgment also influenced the pain empathic responses (Li et al., 2018). Moral judgment is defined as the process of making value judgments about moral behavior, such as what is right or wrong, good or bad, beautiful or ugly (Guglielmo, 2015). As an internal personality trait, moral refers to the kindness and friendliness to others. According to the principle of interpersonal reciprocity in human social relationships (Buunk and Schaufeli, 1999), people who treat others kindly should also be friendly treated. Researchers indeed indicated that people usually showed stronger pain empathic responses to individuals with high moral judgment than those with low moral judgment (Fang et al., 2016; Li et al., 2018). Singer et al. (2006) study also found that participants evoked more pain empathic responses for those who were fair (associated with high moral) to others and showed less empathy for those who were unfair to others.

Both of the facial attractiveness and moral judgment affect the pain empathic responses, however, the effects of facial attractiveness on pain empathy are still in controversial, and how those two factors affect each other remains unknown. The current research conducted two studies to investigate (1) how facial attractiveness affected the perception to other’s pain, and (2) how facial attractiveness and moral judgment affected the pain empathic responses. For study 1, based on previous research, we hypothesized that facial attractiveness would facilitate the pain empathic responses. For study 2, we hypothesized that low facial attractiveness would not decrease the pain empathic responses to people with high moral judgment. Because of that compared with physical appearance, personality characters such as high morality, friendliness and kindness to others have been emphasized by society and education for centuries (Baudson et al., 2016). Morality might be more important than facial attractiveness in the empathetic responses for pain. But whether high facial attractiveness would improve the pain empathic responses to people with low moral judgment would be explored.

## Study 1

### Methods

#### Participants

G*Power 3.1 was used to calculate the sample size required with the parameters set as follows: the effect size f was 0.25, alpha was 0.05, 1-beta was 0.8, the number of groups was 1, the number of times of measurements was 4, and the correlation between repeated measures was 0.5 (Cohen, 1992). Then the minimum sample size required for study 1 was 24. We recruited 29 students at the Southwest University, China. All of them were right-handed and had normal or corrected-to normal vision without history of neurological or psychiatric illnesses. Three participants were excluded from the analysis on account of missing value, reducing the final number to 26 participants (23 females). Their average age was 19.62 years (*SD* = 1.24).

#### Video clips

The process of selecting actors based on attractiveness involves the following steps. Firstly, we published an advertisement on campuses to recruit actors and asked candidates to send us their passport photos. And then five psychology students (three females) selected eight candidates among about 100 candidates based on their physical attractiveness attributes, including symmetry and averageness (Rhodes et al., 2001). The eight actors include two high and two low attractive women with high symmetry and averageness, and two high and two low attractive men with low symmetry and averageness. To check the effectiveness of the selection, we took photos for them in front of the same background and 39 participants (16 males and 23 females) were recruited to rate attractiveness with 9-points Likert according to these photos (Grammer and Thornhill, 1994; Wilson and Eckel, 2006; Andreoni and Petrie, 2008; Deryugina and Shurchkov, 2015; Kantor et al., 2015; Ruffle et al., 2022). Result showed that participants rated the scores of the high facial attractive actors (*M* = 5.21, *SD* = 1.34) significantly higher than those of the low facial attractive actors (*M* = 2.63, *SD* = 1.05), *t*(38) = 9.49, *p* < 0.001, which indicated that the high and low facial attractive level was successfully distinguished.

**FIGURE 1:**
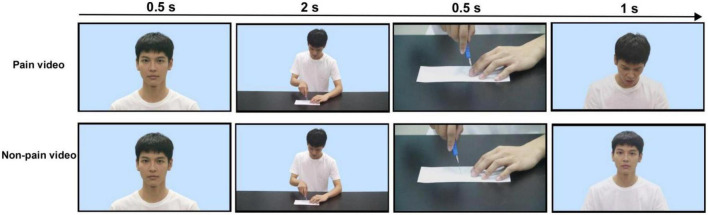
Showed examples of pain and non-pain videos with high attractive male. Each video lasted for 4 s. The first 0.5 s was the actor’s full face photo, followed by 2 s for distant shots, such as the actor cutting paper or fruit, closing a drawer, etc. The next 0.5 s was a close-up shot. In pain conditions the actor performs a painful act (e.g., cutting the finger), in non-pain conditions the actor performs a normal paper-cutting. The last 1 s was the actor’s facial expression. The actor’s face showed pain expression in pain condition, while kept neutral expression all the time in non-pain condition.

The selected actors were invited to act pain and non-pain scenes in daily life. In the video clips, all actors were dressed the same and had their upper bodies taken in front of the same background. Each actor then was filmed in 10 pain and 10 corresponding non-pain scenes in daily life, such as cutting fruit, closing drawer and cutting paper (e.g., Figure 1). All actors were directed by a directing student to perform. And according to Jankowiak-Siuda et al. (2015) study, the selection of recorded stimuli was done by four psychological students based on three key features of expression, such as brow lowering, lid tighten, and upper lip raising for pain scene (Ekman and Friesen, 1978; Kappesser and Williams, 2002). Each video lasts for 4 s. One hundred and sixty video clips in total were initially made.

#### Experimental design and procedure

The design was a 2 (Facial Attractiveness: high, low) × 2 (Pain Type: pain, non-pain) within-subject design. Participants’ pain intensity rating scores of the actors in the video were assessed as the dependent variable.

The experiment was a pain intensity rating task, which was programmed using *PsychoPy* software. Participants were seated at 70 cm directly in front of the computer screen with both eyes at a horizontal and vertical angle of view of less than 6° to the computer screen. The procedure for a single trial of the experiment was as follows. Every video of the actor in pain or non-pain was played first. After watching the video, participants were asked to rate the pain intensity experienced by the actor in the video, with 1 (not painful at all) to 7 (extremely painful) (LaChapelle et al., 2014; Jankowiak-Siuda et al., 2015). Scoring was done by pressing a button and then moving on to the next video. The pain empathy task consisted of two blocks with 160 trials each. One minute break was taken between the two blocks. The experiment took about 30 min to complete. This experiment was approved by the ethics committee of the host institution.

### Data analysis

Participants’ pain intensity rating scores for each actor’s 10 pain videos were recorded. Five videos with higher pain intensity scores of those 10 videos for each actor and the corresponding non-pain videos were chose from the initial data. All the other data were omitted because those pain videos might be less valid to evoke participants’ pain feelings. Therefore, participants’ pain intensity rating scores to 40 pain videos and 40 corresponding non-pain videos were used in the following data analysis. The range of pain ratings for the selected pain videos is from 3.77 to 5.73, and the range for non-pain ones is from 1.00 to 2.19. To test the coefficients of internal consistency of the videos, Cronbach’s coefficient alpha was calculated. Alpha is 0.97 for pain-videos and 0.94 for non-pain videos.

A primary paired sample *t*-test was conducted to examine whether the differences of facial attractiveness were valid. A repeated-measures analysis of variance (ANOVA) was performed, with two within-subject factors, i.e., Facial Attractiveness (high or low), Pain Type (pain or non-pain). An ANOVA on pain intensity ratings was performed by using SPSS 21.0. Bonferroni correction was used for all simple effects analyses when interactions were significant.

### Results

The mean and standard deviations of pain intensity scores to actors with high versus low facial attractiveness under pain and non-pain conditions are shown in Table 1. The main effect of Facial Attractiveness was found, *F*(1, 23) = 10.60, *p* = 0.003, *η_p_*^2^ = 0.34. Paired comparison analyses indicated that participants rated significantly higher pain intensity to actors with high facial attractiveness (*M* = 3.00, *SE* = 0.11) than to actors with low facial attractiveness (*M* = 2.83, *SE* = 0.11). The main effect of Pain Type was significant, *F*(1, 23) = 206.71, *p* < 0.001, *η_p_*^2^ = 0.89. Pairwise comparison analyses indicated that participants rated significantly higher pain intensity under pain condition (*M* = 4.55, *SE* = 0.21) than under non-pain condition (*M* = 1.29, *SE* = 0.07). The interaction of Facial Attractiveness × Pain Type was significant, *F*(1, 23) = 13.16, *p* = 0.001, *η_p_*^2^ = 0.35. Simple effects analyses found that participants rated significantly higher pain intensity to actors with high facial attractiveness than to actors with low facial attractiveness under pain condition (*p* = 0.002). No such facial attractiveness difference was found under non-pain condition (*p* = 0.57).

**TABLE 1:** The mean and standard deviation of pain intensity scores to actors with high versus low facial attractiveness under pain and non-pain conditions.

		Pain	Non-pain
		*M* (*SD*)	*M* (*SD*)
Facial attractiveness	High	4.72 (0.22)	1.30 (0.06)
	Low	4.38 (0.21)	1.28 (0.07)

### Summary

Study 1 investigated the effects of facial attractiveness on participants’ pain empathic responses and proved hypothesis 1. The results found that participants showed higher pain intensity ratings toward high facial attractive actors compared with low facial attractive actors when they experienced pain, which indicated that high facial attractiveness could increase individual’s pain empathic responses. The result was in line with some previous research (e.g., van Leeuwen et al., 2009; Kopiś et al., 2020; Meng et al., 2020). Based on the “beauty is good” theory, people with high facial attractiveness attracted more attention and resulted in more pro-social behavior (Adolphs and Tusche, 2017; Faust et al., 2019; Yang et al., 2020). So attractive actors in pain got higher pain intensity ratings. However, these increasing effects were very limited, for the means differences were very small.

## Study 2

In accordance to existing research, study 1 measured the cognitive pain empathy with pain intensity rating (LaChapelle et al., 2014; Jankowiak-Siuda et al., 2015; Meng et al., 2020). But pain empathy contains two components which are cognitive component and emotional component, so self-uncomfortableness was added in study 2 to measure pain empathy (Goubert et al., 2005). Study 2 explored the effects of facial attractiveness and moral judgment on pain empathy by combining moral stories with those high versus low facial attractive actors in study.

### Methods

#### Participants

G*Power 3.1 was used to calculate the sample size required with the parameters set as follows: the effect size f was 0.25, alpha was 0.05, 1-beta was 0.8, the number of groups was 1, the number of times of measurements was 8, and the correlation between repeated measures was 0.5. The minimum sample size needed was 16. To prevent loss caused by subjects and data missing, 89 participants were recruited at the Southwest University (46 females) for this study in return for 15 RMB. All of them were right-handed and had normal or corrected-to normal vision without history of neurological or psychiatric illnesses. Five participants were excluded from the analysis because they did not complete the experiment, reducing the final number to 84 participants (44 females). Their average age was 19.94 years (*SD* = 1.65).

#### Video clips and morality stories

Those video clips used for the data-analysis in study 1 were used in study 2. The experimenters made up 20 stories each with high or low moral judgments levels (around 150 words). Forty-eight university students (12 males and 36 females) participated in the assessment. Participants were asked to read the stories carefully and to rate the moral level of the characters in the stories on a scale of 1-9, with 1 being very low moral and 9 being very high moral. The four stories with the highest moral level scores (*M* = 8.27, *SD* = 0.13) and the four stories with the lowest moral level scores (*M* = 1.44, *SD* = 0.09) were chosen for this study. The difference in moral level scores between the two groups of stories was significant, *t*(47) = 55.72, *p* < 0.001. To facilitate the participants’ quick identification of the actor’s moral level during the pain empathy task, experimenter extracted synopsis for each moral story and used it in the pain empathy task (around 10 words each). Example of a high moral story synopsis is “Volunteer who undertook to distribute supplies during an epidemic” and example of a low moral story synopsis is “Blackmailer and bully of roommates.” Moral stories and actors were matched with the following principle. Four high moral stories were randomly assigned to two male and two female actors with high or low facial attractiveness, and four low moral stories were randomly assigned to other two male and two female actors with high or low facial attractiveness.

#### Experimental design and procedure

The design was a 2 (Facial Attractiveness: high, low) × 2 (Moral Judgment: high, low) × 2 (Pain Type: pain, non-pain) within-subject three-factor design. The dependent variables were participants’ rating of pain intensity and self-uncomfortableness of the actors in the video. The control variable was the gender of the participants and the actors. Participants were half male and half female and so were the actors.

**FIGURE 2:**
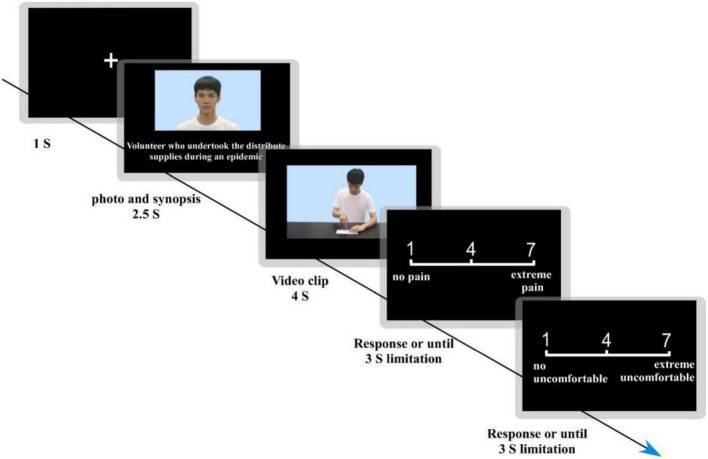
Showed a single trial example. First, a “+” appeared in the center of the computer screen for 1 s, followed by a picture of the actor in the center of the screen for 2.5 s. A summary of the actor’s story was displayed below the picture. A video of the actor in pain or non-pain was then played for 4 s. After watching the video, participants were asked to first rate the pain intensity experienced by the actor in the video, with 1 being not painful at all to 7 being very painful, and then rate the degree of self-uncomfortableness experienced after watching the video, with 1 being not uncomfortable at all and 7 being very uncomfortable.

The experiment was composed of two phases, the first of which involved reading the moral stories of the actors. Participants were firstly shown photographs of eight actors and their stories, as well as an extracted synopsis of each story. Participants were asked to read each actor’s story carefully and familiarize themselves with the synopsis. The second stage was a pain empathy rating task, which was programmed in *PsychoPy* software. Participants were seated at 70 cm directly in front of the computer screen with both eyes at a horizontal and vertical angle of view of less than 6° to the computer screen. The procedure for a single trial of the experiment was as follows. First, a “ + “ appeared in the center of the computer screen for 1 s, followed by a picture of the actor in the center of the screen for 2.5 s. A summary of the actor’s story was displayed below the picture. A video of the actor in pain or non-pain condition was then played for 4 s. After watching the video, 7 points’ rating was adapted to rate the pain empathy (LaChapelle et al., 2014; Jankowiak-Siuda et al., 2015). Participants were asked to rate the pain intensity experienced by the actor in the video, with 1 being not painful at all to 7 being very painful, and then rate the degree of self-uncomfortableness experienced after watching the video, with 1 being not uncomfortable at all and 7 being very uncomfortable. Scorings were done by pressing a button within three seconds limitation and then moving on to the next trial. A single trial example was showed in Figure 2. The pain empathy task consisted of two blocks with 40 randomly presented trials. Each block included five trials for all conditions (i.e., high versus low moral judgment, high versus low facial attractiveness, pain and non-pain). The gender of the actors was also balanced in each block. There was a one minute break between the two blocks. The experiment took about 30 min to complete. This experiment was approved by the ethics committee of the host institution.

### Results

Two repeated-measures analyses of variance (ANOVA) were performed, with three within-subjects factors, i.e., Facial Attractiveness (high or low), Moral Judgment (high or low), and Pain Type (pain or non-pain). ANOVA was performed using SPSS 21.0 for pain intensity and self-uncomfortableness ratings respectively. Bonferroni correction was used for all simple effects analyses when interactions were significant. The mean and standard deviation of pain intensity scores and self-uncomfortableness scores under different facial attractiveness conditions, moral judgments and pain types were shown in Table 2.

**TABLE 2:** The mean and standard deviation of pain empathy scores to actors with high versus low facial attractiveness and high versus low moral judgment under pain and non-pain conditions.

		Pain intensity		Self-uncomfortableness	
		Pain *M* (*SD*)	Non-pain *M* (*SD*)	Pain *M* (*SD*)	Non-pain *M* (*SD*)
High moral	High attractiveness	5.16 (1.02)	1.88 (1.12)	4.29 (1.47)	1.88 (1.11)
	Low attractiveness	5.10 (1.00)	1.85 (1.08)	4.25 (1.37)	1.89 (1.13)
Low moral	High attractiveness	4.83 (1.16)	1.62 (0.93)	2.53 (1.18)	1.68 (0.98)
	Low attractiveness	4.67 (1.16)	1.65 (0.94)	2.43 (1.14)	1.77 (1.17)

#### Pain intensity rating

**FIGURE 3:**
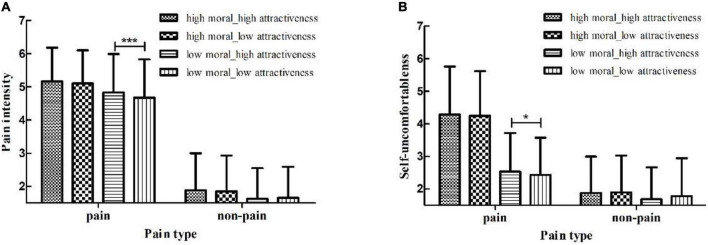
Showed moral judgment and facial attractiveness level differences in **(A)** Pain intensity and **(B)** Self-uncomfortableness under pain versus non-pain conditions for study 2. Participants rated pain intensity and self-uncomfortableness ratings significantly higher for high facial attractiveness actors than for low facial attractiveness actors when they experienced pain and with low moral judgment level. Although no such difference was found for high moral judgment actors, participants indeed rated pain intensity and self-uncomfortableness ratings significantly higher for actors with high moral judgment compared with actors with low moral judgment, regardless of their facial attractiveness. Error Bar represented SD. *p < 0.05, ***p < 0.001.

A significant main effect of Facial Attractiveness was found, *F*(1, 83) = 4.45, *p* = 0.04, *η_p_*^2^ = 0.05. Paired comparison analyses indicated that participants rated pain intensity significantly higher for high facial attractive actors (*M* = 3.37, *SE* = 0.08) than for low facial attractive actors (*M* = 3.32, *SE* = 0.08). The main effect of Moral Judgment was significant, *F*(1, 83) = 30.35, *p* < 0.001, *η_p_*^2^ = 0.27. Paired comparison analyses indicated that participants rated higher pain intensity for actors with high moral (*M* = 3.50, *SE* = 0.08) than those with low moral (*M* = 3.19, *SE* = 0.08). The main effect of Pain Type was significant, *F*(1, 83) = 405.79, *p* < 0.001, *η_p_*^2^ = 0.83. Pairwise comparison analyses indicated that participants rated pain intensity higher in pain condition (*M* = 4.94, *SE* = 0.11) than in non-pain condition (*M* = 1.75, *SE* = 0.11). The interaction of Facial Attractiveness with Pain Type was significant, *F*(1, 83) = 7.18, *p* = 0.009, *η_p_*^2^ = 0.08. Simple effects analyses found that participants rated pain intensity significantly higher for high facial attractive actors (*M* = 4.99, *SE* = 0.11) than for low facial attractive actors (*M* = 4.89, *SE* = 0.11, *p* = 0.001) under pain condition; in non-pain condition, there was no such difference (*p* = 0.96). The interaction between Moral Judgment and Pain Type was significant, *F*(1, 83) = 4.84, *p* = 0.01, *η_p_*^2^ = 0.08. Simple effects analyses found that participants rated high moral actors’ pain intensity (*M* = 5.13, *SE* = 0.11) significantly higher than low moral actors in pain condition (*M* = 4.75, *SE* = 0.13, *p* < 0.001); in non-pain condition, participants also rated high moral actors’ pain intensity significantly higher (*M* = 1.86, *SE* = 0.12) than low moral actors (*M* = 1.64, *SE* = 0.10, *p* < 0.001). The interaction between Facial Attractiveness and Moral Judgment was not significant, *F*(1, 83) = 0.39, *P* = 0.54. However, the interaction between Facial Attractiveness, Moral Judgment, and Pain Type was significant, *F*(1, 83) = 4.04, *p* = 0.05, *η_p_*^2^ = 0.05. Results from simple effects analysis were shown in Figure 3A. For actors with low moral judgment in pain condition, participants rated higher pain intensity ratings to high facial attractive actors (*M* = 4.83, *SE* = 0.13) than to low facial attractive actors, *M* = 4.67, *SE* = 0.13, *p* < 0.001; there were no differences in any of the other conditions (*p* > 0.18).

#### Self-uncomfortableness rating

The main effect of Moral Judgment was significant, *F*(1, 83) = 89.64, *p* < 0.001, *η_p_*^2^ = 0.52. Paired comparison analyses indicated that participants rated their uncomfortableness for high moral actors (*M* = 3.08, *SE* = 0.11) significantly higher than for low moral actors (*M* = 2.10, *SE* = 0.09. Main effect of Pain Type was significant, *F*(1, 83) = 172.99, *p* < 0.001, *η_p_*^2^ = 0.68. Paired comparison analyses indicated that participants rated their self-uncomfortableness significantly higher in pain condition (*M* = 3.37, *SE* = 0.12) than in non-pain condition (*M* = 1.81, *SE* = 0.09). However, the main effect of Facial Attractiveness was not found, *F*(1, 83) = 0.12, *p* = 0.74, *η_p_*^2^ = 0.001. The interaction of Facial Attractiveness and Pain Type was significant, *F*(1, 83) = 5.21, *p* = 0.03, *η_p_*^2^ = 0.06. Simple effects analyses found that in pain condition, participants experienced marginally significant more self-uncomfortableness for high facial attractive actors (*M* = 3.41, *SE* = 0.12) compared to those for low facial attractive actors (*M* = 3.34, *SE* = 0.12, *p* = 0.07); in non-pain condition, no such difference was found (*p* = 0.18). The interaction between Moral Judgment and Pain Type was significant, *F*(1, 83) = 58.27, *p* < 0.001, *η_p_*^2^ = 0.41. Simple effects analyses found that participants rated high moral actors (*M* = 4.27, *SE* = 0.15) significantly higher in pain condition than low moral actors (*M* = 2.48, *SE* = 0.13, *p* < 0.001); in non-pain condition, no such difference was found (*p* = 0.28). The interaction between Facial Attractiveness and Moral Judgment was not significant, *F*(1, 83) = 0.01, *p* = 0.92. Although the interaction between Facial Attractiveness, Moral Judgment, and Pain Type was not significant, *F*(1, 83) = 2.22, *p* = 0.14, we conducted an exploratory simple effects analysis. The similar results pattern in pain intensity was also found for self-uncomfortableness, which was shown in Figure 3B. For actors with low moral judgment in pain condition, participants rated more self-uncomfortableness for high facial attractive actors (*M* = 2.53, *SE* = 0.13) than for low facial attractive actors (*M* = 2.43, *SE* = 0.12, *p* = 0.03). There were no significant differences in any of the other conditions (*p* > 0.13).

### Summary

Study 2 explored the effects of facial attractiveness and moral judgment on pain empathy by filming videos of high and low facial attractive actors in painful and non-painful daily life scene, combined with high and low moral judgment stories. The main finding of the study was that in the high moral judgment with pain condition, no pain intensity ratings and self-uncomfortableness ratings differences between high and low facial attractive actors were found, which proved the hypothesis 2. Low facial attractiveness would not decrease the pain empathic responses for people with high moral. However, in the low moral judgment with pain condition, participants rated the pain intensity and self-uncomfortableness significantly higher for those high facial attractive actors than for those low facial attractive actors. High facial attractiveness elevated participants’ pain empathic responses towards people with low moral judgment.

## General discussion

Study 1 explored the effects of facial attractiveness on pain empathy and showed that facial attractiveness could activate more pain empathetic responses. And then study 2 explored the effects of facial attractiveness and moral judgment on pain empathy, by filming videos of high and low facial attractive actors in pain and non-pain daily life scene, combined with high and low moral judgment stories.

Study 1 found that people showed more empathy responses toward high facial attractive actors under pain condition. The result was consistent with some of the previous research (van Leeuwen et al., 2009; Kopiś et al., 2020; Meng et al., 2020). Based on the influence of “beauty is good” theory, high facial attractive individuals would attract more attention and were thought to have more positive personality qualities, resulting in positive emotional experiences and more pro-social behavior (Adolphs and Tusche, 2017; Faust et al., 2019; Yang et al., 2020). The result was inconsistent with those of Jankowiak-Siuda et al. (2015, 2019) studies. However, the actors in their study were quite few and they measured empathy as the dependent variable rather than pain empathy.

Consistent with previous studies (Fang et al., 2016; Li et al., 2018), study 2 found that participants rated pain intensity and self-uncomfortableness ratings significantly higher for actors with high moral judgment compared with actors with low moral judgment, regardless of their facial attractiveness. Thus, the hypothesis that low facial attractiveness would not decrease the pain empathic responses to people with high moral judgment has been verified. Morality is more important than facial attractiveness in the empathic responses for pain. These increasing effects of moral judgment on pain empathy might be explained by the in-group effects (Turner et al., 1987). People would automatically use moral ratings as a criterion for social categorization. At the same time, they classified individuals with high moral judgment as in-group members. Thus, they developed a positive social identity with the group and produced stronger pain empathic responses to their in-group members (Goldring and Heiphetz, 2020; Chen et al., 2021).

Furthermore, study 2 found that for actors with low moral level in pain, participants rated pain intensity and self-uncomfortableness ratings significantly higher to those with high facial attractiveness compared to those with low facial attractiveness. These results indicated that the advantageous effect of facial attractiveness was only found in those with low moral judgment. Previous research has found that high-facial-attractiveness defendants were considered less guilty at trial and received less severe punishment than low-facial-attractiveness defendants (Efrain, 1974; Beaver et al., 2019; Bitton and Zvi, 2019; Petrauskaitė and Čunichina, 2019; Klein and Shtudiner, 2021). When low moral actors are subjected to painful stimuli, participants may be more forgiving and felt less aversive toward high facial attractive actors, and therefore showed stronger empathic responses. However, it is noteworthy that those differences were very small, which indicated that the increasing effects of facial attractiveness for actors with low moral were very limited.

The results confirmed the cognitive model of empathy (Goubert et al., 2005). The model stated that the development of empathy experienced two stages, i.e., emotional stage and cognitive stage. Facial attractiveness affected the emotional stage and moral judgment affected the cognitive stage. Although high facial attractiveness elicited a certain stronger empathy at the emotional stage, moral information in the subsequent cognitive evaluation stage affected the final empathy responses. Thus, actors with high facial attractiveness firstly elicited stronger empathic responses in the emotional stage compared to actors with low facial attractiveness. However, in the follow-up stage, the low morality of the character diminished the pain empathy level. Thus, only actors with high moral judgment were received high pain empathic responses.

The contribution of this study is that for the first time to explore the interaction between facial attractiveness and moral judgment in empathic responses to pain. The results showed that morality is more important than facial beauty in triggering people’s benevolence. For example, a person with a heart of gold, regardless of his physical attractive level, would always get sympathy when he was suffering and would therefore had more opportunities to get help. People should focus less on physical beauty but more on moral improvement. So this study has critical social significance.

Furthermore, the experimental materials have good ecological validity and could evoke pain empathic response better. Previous study used materials with low ecological validity which placed needles on the face with neutral expression, i.e., injections in the face, as the pain material, and cotton swabs as the control material (Meng et al., 2020). In this study, pain videos in daily life were created as the experimental materials. Participants were able to see pain events, pain facial expressions and facial attractiveness at the same time. Although the study by Jankowiak-Siuda et al. (2015) also used video materials of common pain scenes from daily life, the materials contained only one actor in each condition. We doubled the actors and the content and structure of the videos were standardized. Moreover, the procedure of the task was improved. For each trial in the task, information about the actor’s face and a summary of the moral story were presented firstly, which could fully evoked participants’ observation of the actor’s face and moral appraisal.

However, this study also has some limitations. Firstly, we didn’t balance the gender in study 1. But we improved this in study 2 and found similar result. Furthermore, the sample of university students used in this study was limited and lacked evidence from a wider sample to support it. Future research should further test whether the results are generalized in other samples such as the working population and other age groups. Secondly, as a behavioral study, it is difficult for this study to examine the stages of pain empathy influenced by facial attractiveness and moral judgment and their neural mechanisms. Previous studies found that facial attractive mainly affected the early components of ERP (Kopiś et al., 2020) and moral judgment mainly affected the late components of ERP (Li et al., 2018). The neurophysiologic mechanisms about how these two factors affect pain empathy differently should be further investigated in future studies. In addition, future research could further explore how facial attractiveness and moral judgment influence people’s other pro-social behaviors, such as financial donations or social acceptance.

The main findings of this study were that when facial attractiveness conflicted with moral judgment, for actors with low moral level, high facial attractiveness enhanced people’s pain empathic responses, but this advantage of facial attractiveness was very limited. For high moral actors, low facial attractiveness did not reduce people’s pain empathic responses. Moral judgment is a more important factor in promoting pain empathy than facial attractiveness. In daily life, people could improve their moral integrity to promote pain empathic responses and pro-social behaviors.

## Data availability statement

The raw data supporting the conclusions of this article will be made available by the authors, without undue reservation.

## Ethics statement

The studies involving human participants were reviewed and approved by Institutional Review Board of Faculty of Psychology, Southwest University. The patients/participants provided their written informed consent to participate in this study. Written informed consent was obtained from the individual(s) for the publication of any potentially identifiable images or data included in this article.

## Author contributions

ZY developed the research design and wrote the manuscript. JZ developed the research design, prepared the experimental materials, and analyzed the data. All authors collected the data and approved the final version of the manuscript for submission.

## References

[B1] Adolphs R. Tusche A. (2017). From faces to prosocial behavior: Cues, tools, and mechanisms. *Curr. Dir. Psychol. Sci.* 26 282–287. 10.1177/0963721417694656 28943722 PMC5608038

[B2] Andreoni J. Petrie R. (2008). Beauty, gender and stereotypes: Evidence from laboratory experiments. *J. Econ. Psychol.* 29 73–93. 10.1016/j.joep.2007.07.008

[B3] Balconi M. Kopis N. Angioletti L. (2020). Does aesthetic judgment on face attractiveness affect neural correlates of empathy for pain? A fNIRS study. *Exp. Brain Res.* 238 2067–2076. 10.1007/s00221-020-05867-y 32638037

[B4] Baudson T. G. Weber K. E. Freund P. A. (2016). More than only skin deep: Appearance self-concept predicts most of secondary school students’ self-esteem. *Front. Psychol.* 7:1568. 10.3389/fpsyg.2016.01568 27803681 PMC5067372

[B5] Beaver K. M. Boccio C. Smith S. Ferguson C. J. (2019). Physical attractiveness and criminal justice processing: Results from a longitudinal sample of youth and young adults. *Psychol. Law* 26 669–681. 10.1080/13218719.2019.1618750 31984103 PMC6762156

[B6] Bitton M. S. Zvi L. (2019). Chivalry and attractiveness bias in police officer forensic judgments in Israel. *J. Soc. Psychol.* 159 503–517. 10.1080/00224545.2018.1509043 30152730

[B7] Buunk B. P. Schaufeli W. B. (1999). Reciprocity in interpersonal relationships: An evolutionary perspective on its importance for health and well-being. *Eur. Rev. Soc. Psychol.* 10 259–291. 10.1080/14792779943000080

[B8] Chen J. Li Z. Lv Y. F. Li C. L. Wang Y. Wang R. R. (2015). Empathy for pain: A novel bio-psychosocial-behavioral laboratory animal model. *Acta Physiol. Sin.* 67 561–570. 26701631

[B9] Chen J. Wu K. Shi Y. P. Ai X. Q. (2021). The relationship between dispositional self-construal and empathy for ingroup and outgroup members’ pain: Evidence from ERPs. *Acta Psychol. Sin.* 53 629–638. 10.3724/SP.J.1041.2021.00629

[B10] Chen J. Zhong J. Zhang Y. Li P. Zhang A. Tan Q. (2012). Electrophysiological correlates of processing facial attractiveness and its influence on cooperative behavior. *Neurosci. Lett.* 517 65–70. 10.1016/j.neulet.2012.02.082 22410307

[B11] Ch’ng K. S. (2021). Attractiveness, trust and trustworthiness: An experimental study. *Malays. J. Econ. Stud.* 58 45–57. 10.22452/MJES.vol58no1.3

[B12] Cohen J. (1992). Statistical power analysis. *Curr. Dir. Psychol. Sci.* 1 98–101. 10.1111/1467-8721.ep10768783

[B13] Danziger N. Prkachin K. M. Willer J. C. (2006). Is pain the price of empathy? The perception of others pain in patients with congenital insensitivity to pain. *Brain* 129 2494–2507. 10.1093/brain/awl155 16799175

[B14] Deryugina T. Shurchkov O. (2015). Now you see it, now you don’t: The vanishing beauty premium. *J. Econ. Behav. Organ.* 116 331–345. 10.1016/j.jebo.2015.05.007

[B15] Dion K. Berscheid E. Walster E. (1972). What is beautiful is good. *J. Pers. Soc. Psychol.* 24 285–290. 10.1037/h0033731 4655540

[B16] Efrain M. G. (1974). The effect of physical appearance on the judgment of guilt, interpersonal attraction, and severity of recommended punishment in a simulated jury task. *J. Res. Pers.* 8 45–54. 10.1016/0092-6566(74)90044-0

[B17] Ekman P. Friesen W. V. (1978). *Facial action coding system: Investigatoris guide.* Palo Alto, CA: Consulting Psychologists Press.

[B18] Fang C. Ning M. Luo Y. J. (2016). Moral judgment modulates neural responses to the perception of other’s pain: An ERP study. *Sci. Rep.* 6:20851. 10.1038/srep20851 26865250 PMC4749990

[B19] Faust N. T. Chatterjee A. Christopoulos G. I. (2019). Beauty in the eyes and the hand of the beholder: Eye and hand movements’ differential responses to facial attractiveness. *J. Exp. Soc. Psychol.* 85:103884. 10.1016/j.jesp.2019.103884

[B20] Giummarra M. J. Fitzgibbon B. M. Georgiou-Karistianis N. Beukelman M. Verdejo-Garcia A. Blumberg Z. (2015). Affective, sensory and empathic sharing of another’s pain: The empathy for pain scale. *Eur. J. Pain* 19 807–816. 10.1002/ejp.607 25380353

[B21] Gladstein G. A. (1983). Understanding empathy: Integrating counseling, developmental, and social psychology perspectives. *J. Couns. Psychol.* 30 467–482. 10.1037/0022-0167.30.4.467

[B22] Goldring M. R. Heiphetz L. (2020). Sensitivity to ingroup and outgroup norms in the association between commonality and morality. *J. Exp. Soc. Psychol.* 91:104025. 10.1016/j.jesp.2020.104025

[B23] Goubert L. Craig K. D. Vervoort T. Morley S. Sullivan M. J. L. de Williams C. A. C. (2005). Facing others in pain: The effects of empathy. *Pain* 118 285–288. 10.1016/j.pain.2005.10.025 16289804

[B24] Grammer K. Thornhill R. (1994). Human (*Homo sapiens*) facial attractiveness and sexual selection: The role of symmetry and averageness. *J. Comp. Psychol.* 108 233–242. 10.1037/0735-7036.108.3.233 7924253

[B25] Guglielmo S. (2015). Moral judgment as information processing: An integrative review. *Front. Psychol.* 6:1637. 10.3389/fpsyg.2015.01637 26579022 PMC4626624

[B26] Jackson P. L. Rainville P. Decety J. (2006). To what extent do we share the pain of others? Insight from the neural bases of pain empathy. *Pain* 125 5–9. 10.1016/j.pain.2006.09.013 16997470

[B27] Jankowiak-Siuda K. Duszyk A. Dopierała A. Bujwid K. Rymarczyk K. Grabowska A. (2019). Empathic responses for pain in facial muscles are modulated by actor’s attractiveness and gender, and perspective taken by observer. *Front. Psychol.* 10:624. 10.3389/fpsyg.2019.00624 30949111 PMC6437081

[B28] Jankowiak-Siuda K. Rymarczyk K. Żurawski Ł Jednoróg K. Marchewka A. (2015). Physical attractiveness and sex as modulatory factors of empathic brain responses to pain. *Front. Behav. Neurosci.* 9:236. 10.3389/fnbeh.2015.00236 26441569 PMC4561342

[B29] Kantor J. Shapir O. M. Shtudiner Z. (2015). Beauty is in the eye of the employer: Labor market discrimination of accountants. *Front. Psychol.* 13:928451. 10.3389/fpsyg.2022.928451 35967655 PMC9372560

[B30] Kappesser J. Williams A. C. (2002). Pain and negative emotions in the face: Judgements by health care professionals. *Pain* 99 197–206. 10.1016/s0304-3959(02)00101-x 12237197

[B31] Klein G. Shtudiner Z. (2021). Judging severity of unethical workplace behavior: Attractiveness and gender as status characteristics. *Bus. Res. Q.* 24 19–33. 10.1177/2340944420916100

[B32] Kogler L. Müller V. I. Werminghausen E. Eickhoff S. B. Derntl B. (2020). Do I feel or do I know? Neuroimaging meta-analyses on the multiple facets of empathy. *Cortex* 129 341–355. 10.1016/j.cortex.2020.04.031 32562973 PMC7390692

[B33] Kopiś N. Francuz P. Zabielska-Mendyk E. Augustynowicz P. (2020). Feeling other people’s pain: An event-related potential study on facial attractiveness and emotional empathy. *Adv. Cogn. Psychol.* 16 169–175. 10.5709/acp-0294-8 32685061 PMC7355155

[B34] LaChapelle D. L. Lavoie S. Higgins N. C. Hadjistavropoulos T. (2014). Attractiveness, diagnostic ambiguity, and disability cues impact perceptions of women with pain. *Rehabil. Psychol.* 59 162–170. 10.1037/a0035894 24611920

[B35] Li X. Huang Y. Luo Y. Li H. Shi K. (2018). Is good person more pitiful? Moral judgment influences the empathy for other’s pain: An ERP study. *Chin. J. Clin. Psychol.* 26 47–51+73.

[B36] Meng J. Li X. Peng W. Li Z. Shen L. (2020). The interaction between pain and attractiveness perception in others. *Sci. Rep.* 10:5528. 10.1038/s41598-020-62478-x 32218469 PMC7099075

[B37] Ong D. (2022). The college admissions contribution to the labor market beauty premium. *Contemp. Econ. Policy* 40 491–512. 10.1111/coep.12570

[B38] Petrauskaitė G. Čunichina K. (2019). Effects of a perpetrator’s physical attractiveness, socioeconomic status and gender on behaviour perception of the participants of sexual harassment situations. *Psichologija* 60 58–71. 10.15388/Psichol.2019.9

[B39] Rhodes G. Yoshikawa S. Clark A. Lee K. McKay R. Akamatsu S. (2001). Attractiveness of facial averageness and symmetry in non-western cultures: In search of biologically based standards of beauty. *Perception* 30 611–625. 10.1068/p3123 11430245

[B40] Ruffle B. J. Sherman A. Shtudiner Z. (2022). Gender and beauty price discrimination in produce markets. *J. Behav. Exp. Econ.* 97:101825. 10.1016/j.socec.2022.101825

[B41] Shamay-Tsoory S. G. (2011). The neural bases for empathy. *Neuroscientist* 17 18–24. 10.1177/1073858410379268 21071616

[B42] Shtudiner Z. (2020). The impact of attractiveness on employability: Gender differences in peer effects. *Manage. Decis. Econ.* 41 1613–1620. 10.1002/mde.3207

[B43] Shtudiner Z. Klein G. (2020). Gender, attractiveness, and judgment of impropriety: The case of accountants. *Eur. J. Polit. Econ.* 64:101916. 10.1016/j.ejpoleco.2020.101916

[B44] Singer T. Seymour B. O’Doherty J. P. Stephan K. E. Dolan R. J. Frith C. D. (2006). Empathic neural responses are modulated by the perceived fairness of others. *Nature* 439 466–469. 10.1038/nature04271 16421576 PMC2636868

[B45] Turner J. C. Hogg M. A. Oakes P. J. Reicher S. D. Wetherell M. S. (1987). *Rediscovering the social group: A theory of self-categorization.* Oxford: Basil Blackwell.

[B46] van Leeuwen M. L. Veling H. van Baaren R. B. Dijksterhuis A. (2009). The influence of facial attractiveness on imitation. *J. Exp. Soc. Psychol*. 45 1295–1298. 10.1016/j.jesp.2009.07.008

[B47] Wilson R. K. Eckel C. C. (2006). Judging a book by its cover: Beauty and expectations in the trust game. *Polit. Res. Q.* 59 189–202. 10.1177/106591290605900202

[B48] Yang X. J. Deng X. Y. Bhadauria A. (2020). Does mere exposure to beauty-related words promote prosocial behavior? Exploring the mental association between beauty and prosociality. *J. Assoc. Consum. Res.* 5 106–116. 10.1086/706509

